# Highly Expressed CYBRD1 Associated with Glioma Recurrence Regulates the Immune Response of Glioma Cells to Interferon

**DOI:** 10.1155/2021/2793222

**Published:** 2021-07-16

**Authors:** Mingjie Qing, Jiahao Zhou, Weijian Chen, Lijuan Cheng

**Affiliations:** ^1^Department of Clinical Medicine, College of Medicine, Hunan University of Chinese Medicine, Changsha, Hunan 410208, China; ^2^Department of Neurology, The Third Xiangya Hospital of Central South University, Changsha, Hunan 410013, China; ^3^Department of Pathology, Hunan Children's Hospital, Changsha, Hunan 410007, China; ^4^Department of Biochemistry and Molecular Biology, Hunan University of Chinese Medicine, Changsha, Hunan 410013, China

## Abstract

Invasiveness, resistance to treatment, and recurrence of gliomas are significant hurdles to successful treatment regimens. Data sets from Gene Expression Omnibus (GEO), CGGA-RNAseq, and The Cancer Genome Atlas Glioblastoma Multiforme (TCGA-GBM) were analyzed, and an increased expression of Cytochrome B Reductase 1 (CYBRD1) was identified and could be associated with aggravated clinical outcomes. Gene ontology (GO) enrichment analysis indicated that CYBRD1 co-expressed genes are enriched during an immune response. CYBRD1 overexpression in glioma cell lines is enhanced, whereas CYBRD1 silencing attenuated the aggressiveness of glioma cells. In IFN-*α*-treated glioma cells, IFN-*α* suppressed the viability and migratory ability and invasive ability of glioma cells, whereas CYBRD1 overexpression attenuated the antitumor effects of IFN-*α*. CYBRD1 could potentially serve as a biomarker for glioma recurrence. CYBRD1 overexpression enhances glioma cell aggressiveness and attenuates glioma cell response to IFN-*α*.

## 1. Introduction

Gliomas are the most common primary cerebral tumors in adults [1, 2]. The impaired prognosis rates in high-grade glioma and in recurring glioma patients account for the high fatality numbers worldwide [3, 4]. Despite the alarming numbers, the molecular pathogenetic mechanisms of gliomas remain unclear as of yet.

Glioblastomas are the most aggressive type of glioma due to high relapse rates following resection. Furthermore, the standard treatment regimen includes invasive procedures such as maximal surgical resection and radiotherapy with concomitant temozolomide chemotherapy [5–8]. Despite the aggressiveness of the treatment regimen, the median survival of patients having undergone surgery was a mere 14 months with a 5-year survival rate of less than 10%. This poor prognosis can be attributed to the invasive abilities of glioblastomas coupled with their reliance on treatment. [9]. Furthermore, most patients diagnosed with glioblastomas suffer from recurring tumors despite initial aggressive treatment [10, 11]. In addition to well-established treatment regimens, evidence suggests that immunotherapy can aid glioma treatment [12, 13]. For example, Th1 cytokines, including IFN (interferon), IL (interleukin)-2, and IL-12, can enhance cell-mediated immunity and play an antitumor role [14]. IFN-*β* and IFN-*α* exert antiproliferative functions through inducing p53, activating CD8(+) T-lymphocytes and macrophages, secreting chemokines, and downregulating miR-21, thus playing an antitumor role [15]. An in-depth comprehension of the underlying pathway of glioma carcinogenesis could potentially provide promising neo-adjuvant effects and improve the overall therapeutic effect.

Numerous pathways have been found to contribute to glioma development, including EGFR gene amplification, PTEN gene mutations, and TP53 in different frequencies [16], and the malignant progression of human gliomas involves a sequential accumulation of genetic changes. The identification and isolation of genes and proteins obtaining the biomarker potentials and playing a crucial role in tumorigenesis [17] would undoubtedly invigorate in the quest for a successful glioma treatment regimen.

Cytochrome B reductase 1 (CYBRD1), also known as duodenal cytochrome B (Dcytb), is an enzyme encoded by the CYBRD1 gene. CYBRD1 was first identified as an iron reductase, which can catalyze the reduction of Fe^3+^ required for the duodenum of mammals to absorb dietary iron to Fe^2+^ [18]. Cancer cells exhibit an enhanced requirement for iron compared to normal cells; thus, CYBRD1 has been reported to play a critical role in carcinogenesis. Willis et al. regarded CYBRD1 as one of the twelve genes with high mRNA expression and as being prognostic of poor outcome in patients suffering from ovarian cancer [19]. Boult et al. [20] reported that CYBRD1 was overexpressed during the development of Barrett's metaplasia to adenocarcinoma. Brookes et al. [21] confirmed that increased expression of iron importin (including CYBRD1) is associated with the progression of colorectal cancer. Higher CYBRD1 expression predicted prolonged metastasis-free and relapse-free survival in patients suffering from breast cancer [22]. Higher levels of CYBRD1 in recurring or high-grade gliomas suggest its role in carcinogenesis and development.

Data sets from the Gene expression Omnibus (GGNA-RNAseq) and The Cancer Genome Atlas glioblastoma multiforme (TGCA-GBM) were analyzed to identify the genes upregulated in recurring and high-grade gliomas. Following identification, the correlation of these genes with the survival (relapse-free and overall survival) of glioma patients was analyzed based on TGCA-GBMLGG. Furthermore, the specific effects of CYBRD1 upon the phenotype of glioma cells were subsequently determined. Following this, CYBRD1 co-expression genes were identified and used in a GO (Gene Ontology) enrichment analysis. The CYBRD1 co-expression genes showed to be enriched within interferon response pathways. Therefore, the effects of CYBRD1 on interferon-stimulated glioma cells were examined.

## 2. Materials and Methods

### 2.1. Clinical Sampling

A total of 18 primary glioma tissue samples of different grades (WHO II, *n* = 6; WHO III, *n* = 6; WHO IV, *n* = 6) and 6 noncancerous peritumoral brain edema (PTBE) tissues (nonglioma normal tissues) were harvested from glioma patients having undergone surgical resection at the Third Xiangya Hospital of Central South University. All the samples have been diagnosed and confirmed through pathological analysis. Following surgical resection, all tissues were immediately stored at −80^o^C pending protein and RNA extraction or alternatively prepared as paraffin-embedded blocks pending immunochemistry analysis. The clinical sampling was performed with the approval of the Review Boards of the Third Xiangya Hospital of Central South University and conducted as per the principles of the Declaration of Helsinki. Written informed consent was obtained from each patient enrolled.

### 2.2. Bioinformatics Analysis

To identify genes upregulated in high-grade or recurrent gliomas, data sets from GEO, CGGA-RNAseq, and TCGA-GBM were analyzed using the integrated bioinformatics method and Robust Rank Aggregation (RRA) [23]. Differentially expressed genes between high-grade or recurrent gliomas in GSE4271, GSE58399, GSE60898, GSE62153, and GSE7696 were analyzed using the RRA method. Then, the GBMLGG expression matrix and clinical information (*n* = 695) in the Xena database (https://xenabrowser.net/datapages/) were downloaded, and the univariate Cox-regression analysis was performed using the R language toolkit survival and survminer.

### 2.3. Cell Resource

Human glioma cell line LN229 (CRL-2611™) was obtained from ATCC and cultured in DMEM (ATCC) supplemented with 10% FBS (Invitrogen). Glioblastoma cell line T98G (ATCC® CRL-1690™) was procured from ATCC (Manassas, VA, USA) and cultured in EMEM (Invitrogen) supplemented with 10% FBS (Invitrogen). All the cells were cultured at 37°C in 5% CO_2_. For interferon treatment, cells were stimulated with IFN-*α* (2000 U/ml, Sangon Biotech, China) for 24 h followed by other experimental investigations.

### 2.4. Cell Transfection

CYBRD1 overexpression was achieved by transfecting pcDNA3.1/CYBRD1 OE, and CYBRD1 silencing was performed by transfecting small interfering RNA targeting CYBRD1 (si-NC/si-CYBRD1). pcDNA3.1 or si-NC was used as a negative control (all the plasmid and siRNA were obtained from GenePharma, China). All transfections were performed using Lipofectamine 3000 Reagent (Thermo Fisher Scientific, Waltham, MA, USA).

### 2.5. Histopathological Analysis by H&E Staining

Tissue samples were fixed in 4% paraformaldehyde, embedded in paraffin, and cut into 4-*μ*m thick sections. H&E staining was performed to observe the histopathological features [24]. At least 5 fields were analyzed upon each section, and images were captured by an Olympus microscope.

### 2.6. Immunohistochemical Staining

Tissue sections were deparaffinized and rehydrated, and antigen retrieval was performed. Then, sections were used for IHC staining with anti-CYBRD1 (HPA014757; Sigma-Aldrich, St. Louis, MI, USA) as per the instructions manual of VectaStain Universal ABC kit (Vector Laboratories, Burlingame, CA, USA). The slides were counterstained with hematoxylin. Finally, the sections were observed under a microscope, and representative images were photographed.

### 2.7. Real-Time Quantitative Polymerase Chain Reaction (RT-qPCR)

RT-qPCR was performed to detect the relative mRNA level. Total RNA was extracted from tissues or cell lines using TRIzol reagent as per protocol. cDNA was generated with 1 *μ*g total RNA using MMLV reverse transcriptase (Promega, Madison, WI, USA) and random primers. GAPDH was used as an endogenous control. The fold change for each target gene relative to the control group was calculated using the 2^−ΔΔCt^ method.

### 2.8. Immunoblotting

RIPA buffer (Beyotime, Shanghai, China) with protease inhibitor was used to extract protein from tissue samples or cell lines. BCA quantitative assay (Beyotime) was used to measure the protein concentration. A total of 20 mg protein was separated by Tris-glycine SDS-PAGE (4–12%; Invitrogen) and then transferred onto a polyvinylidene fluoride (PVDF) membrane (Millipore, Burlington, MA, USA). Blocking was performed by using 5% BSA for 2 h at room temperature. The membrane was incubated with primary antibodies overnight at 4°C. The following antibodies were used anti-CYBRD1 (HPA014757, Sigma-Aldrich), anti-I*κ*B*α* (ab32518, Abcam, Cambridge, MA, USA), anti-p-STAT3 (ab267373, Abcam), anti-STAT3 (10253-2-AP, Proteintech, Wuhan, China), and anti-GAPDH (T0004, Affinity Biosciences, Cincinnati, OH, USA). Then, the membranes were incubated with the secondary antibodies (Cowin Biotech Co, Beijing, China) for 2 h at room temperature. Protein accumulation was detected by Western-light Chemiluminescent Detection System (Peiqing, Shanghai, China).

### 2.9. CCK-8 Assay for Cell Viability

A cell viability examination was performed using a CCK-8 kit (Beyotime, Shanghai, China). Cells were seeded into 96-well plates at a density of 5 × 10^3^ cells/well. Two hours before the detection, 20 *μ*l of CCK-8 solution was added to each well, followed by incubation at 37°C. The optical density (OD) value was determined at a wavelength of 450 nm on a microplate reader.

### 2.10. Wound Healing for Cell Migration

The cells were respectively digested, and the cell concentration was adjusted to 5 × 10^5^ cells/ml. Cell suspensions (100 *μ*l) were plated in 96-well plates coated with Matrigel, routinely cultured until the cell monolayer emerged. Then a cell scratch test was performed. The cells were cultured in DMEM or EMEM supplemented with 1% FBS, and the scratch area was measured under a microscope. The cells were treated with 1 *μ*g/ml mitomycin C (Sigma, USA) for 1 h and then allowed to culture for another 24 h in a complementary medium, and then the relative distance of cell migration to injury area was equally measured under a microscope.

### 2.11. Transwell for Cell Invasion

The invasion capacity was determined using polycarbonate Transwell filters following the aforementioned methods [25]. The serum-deprived medium was used in the upper chambers coated with Matrigel, and the medium containing the serum was used in the lower chambers. After discarding the noninvasive cells in the top chambers, the invasive cells on the lower membrane surface were fixed and stained with crystal violet (Beyotime Institute of Biotechnology, Haimen, China) for nuclear staining. The number of cells was counted under a microscope.

### 2.12. Xenograft Tumor Models in Mice

Eighteen male nude BALB/C mice (4–5 weeks old) were purchased from the SLAC Laboratory Animal Company (Hunan, China). All animal experiments were approved by the Ethics Committee of Third Xiangya Hospital of Central South University. The subcutaneous tumor model is established via subcutaneous injection of 2 × 10^6^ LN229 cells (100 *μ*l) into the right flanks of mice. At day 8, mice-bearing tumors of approximately 50 mm^3^ in volume were randomly divided into 3 groups: (a) intratumoral injection of PBS (PBS group); (b) intratumoral injection of IFN-*α* (5 × 10^4^ U/mouse/day) (IFN-*α* group); (c) intratumoral injection of IFN-*α* (5 × 10^4^ U/mouse/day), and CYBRD1 overexpression lentivirus (2 × 10^6^ TU/mice) (IFN-*α*+CYBRD1 OE group). At day 28, mice were sacrificed, and the tumors were collected.

### 2.13. Statistical Analysis

Results from at least three independent experiments were processed using GraphPad and then expressed as means ± SD. Data were statistically analyzed by one-way analysis of variance (ANOVA) followed by Tukey's multiple comparison test or independent sample *t*-test. A *p*-value of <0.05 was deemed as statistically significant.

## 3. Results

### 3.1. CYBRD1 Expression in Tissue Samples according to the Online Database

The differentially expressed genes between high-grade or recurrent gliomas in GSE62153, GSE4271, GSE58399, GSE60898, and GSE7696 were intersected in a total of 36 upregulated genes and 26 downregulated genes, as depicted by the hierarchical clustering heatmap (Figure S1(a)). Fifty one of the 62 differentially expressed genes were found to be related to the overall survival of glioma patients. Among them, 26 are risk factors (hazard ratio> 1, *p* value <0.05) and 32 are protective factors (hazard ratio <1, *p* value <0.05). Among the 26 risk factors, 22 were the upregulated genes obtained from the first step (Figures S1(b) and S1(c)). The top 4 upregulated genes with a hazard ratio of >1.5 were chosen, and the expression of these 4 genes was examined in our collected normal and tumor tissues. As shown by the RT-PCR analysis, only CYBRD1 mRNA levels were significantly upregulated in glioma tissues compared to the nonglioma normal tissues (Figure S1(d)). As a result, CYBRD1 was selected for further experiments.

**Figure 1:**
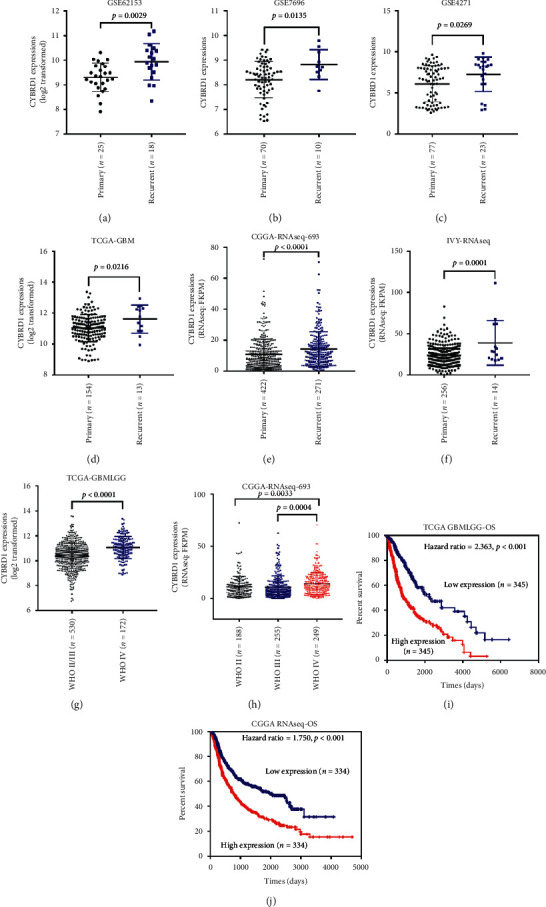
CYBRD1 expression in tissue samples according to an online database. (a) CYBRD1 expression in 25 primary glioma tissues and 18 recurrent glioma tissues, according to GSE62153. (b) CYBRD1 expression in 70 primary glioma tissues and 10 recurrent glioma tissues, according to GSE7696. (c) CYBRD1 expression in 77 primary glioma tissues and 23 recurrent glioma tissues, according to GSE4271. (d) CYBRD1 expression in 154 primary glioma tissues and 13 recurrent glioma tissues, according to TCGA-GBM. (e) CYBRD1 expression in 422 primary glioma tissues and 271 recurrent glioma tissues, according to CGGA-RNAseq-693. (f) CYBRD1 expression in 256 primary glioma tissues and 14 recurrent glioma tissues, according to IVY-RNAseq. (g) CYBRD1 expression in 530 WHO II/III glioma cases and 172 WHO IV glioma cases, according to TCGA-GBMLGG. (h) CYBRD1 expression in 188 WHO II glioma cases, 255 WHO III glioma cases, and 249 WHO IV glioma cases, according to CGGA-RNAseq-693. (i) Cases from TCGA-GBMLGG were grouped by the median value of CYBRD1 expression into the high and low CYBRD1 groups. The correlation of CYBRD1 expression and the overall survival in patients with glioma was analyzed using a Cox proportional hazards model and Log Rank test, and the results were shown as Kaplan–Meier curves. (j) Cases from CGGA-RNAseq were grouped by the median value of CYBRD1 expression into the high and low CYBRD1 groups. The correlation of CYBRD1 expression and the overall survival in patients with glioma was analyzed using a Cox proportional hazards model and Log Rank test, and the results were shown as Kaplan–Meier curves.

Online data mining was performed to confirm the expression status of CYBRD1 during glioma development. According to the data from GSE62153, GSE7696, and GSE4271, CYBRD1 expression was upregulated in recurrent glioma tissues compared to primary glioma tissue samples (Figures 1(a)–1(c)). According to TCGA-GBM, CYBRD1 expression was upregulated in recurrent glioma tissues (*n* = 13) compared to those in primary glioma tissues (*n* = 154; Figure 1(d)). According to CGGA-RNAseq-693, CYBRD1 expression was upregulated in recurrent glioma tissues compared to those in primary glioma tissues (Figure 1(e)). According to IVY-RNAseq, CYBRD1 expression was upregulated in recurrent glioma tissues compared with that in primary glioma tissues (Figure 1(f)). The online data indicate that CYBRD1 expression is abnormally upregulated in recurrent glioma tissues, suggesting that it could be considered a marker that is possibly involved in glioma recurrence.

### 3.2. Glioma Patients with Higher CYBRD1 Expression Predict a Poorer Prognosis

The potential of CYBRD1 as a biomarker for glioma prognosis was subsequently examined using online data. According to TCGA-GBMLGG, the expression of CYBRD1 showed to be dramatically upregulated within high-grade glioma tissue samples compared with low-grade glioma tissue samples (Figure 1(g)). According to CGGA-RNAseq-693, CYBRD1 expression was significantly upregulated in WHO IV glioma cases compared with that in WHO II glioma cases and WHO III glioma cases Figure 1(h)). According to the Kaplan–Meier curves, higher CYBRD1 expression was associated with poorer overall survival in patients with glioma based on TCGA-GBMLGG (Figure 1(i)). Similarly, Figure 1(j) shows that glioma patients with higher CYBRD1 expression predicted poorer survival based on CGGA-RNAseq. It is therefore suggested that higher CYBRD1 expression predicts a poorer prognosis in glioma patients.

### 3.3. Expression and Protein Levels of CYBRD1 in Clinical Tissue Samples

**Figure 2:**
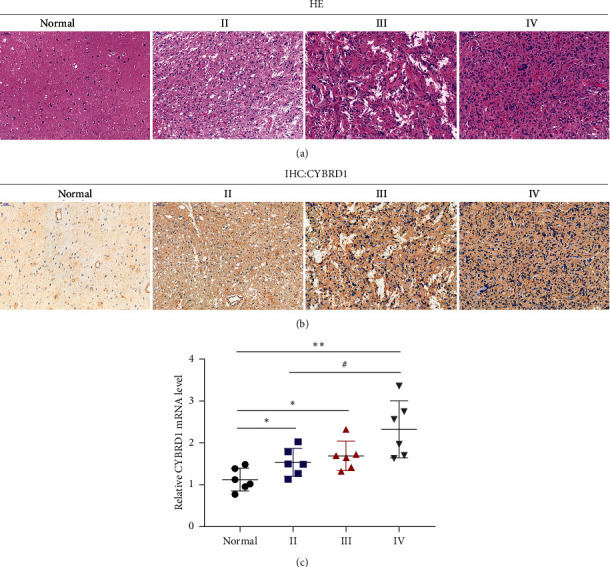
Expression and protein levels of CYBRD1 in clinical tissue samples. (a) The histopathological features of glioma tissues (WHO II/III/IV) and nonglioma normal tissues were examined by H&E staining. (b) The protein content and distribution of CYBRD1 were examined in glioma tissues (WHO II/III/IV) and nonglioma normal tissues by immunohistochemical (IHC) staining. (c) The mRNA expression levels of CYBRD1 were examined in glioma tissues (WHO II, *n* = 6; WHO III, *n* = 6; WHO IV, *n* = 6) and nonglioma normal tissues (*n* = 6) by RT-qPCR. ^*∗*^*p* < 0.05, ^∗∗^*p* < 0.01, ^#^*p* < 0.05.

Glioma samples of different grades and nonglioma normal samples were collected and confirmed for the histopathological features through H&E staining (Figure 2(a)). Then, the protein content and distribution of CYBRD1 were subsequently examined in glioma tissues (WHO II/III/IV) and nonglioma normal tissues by IHC staining, as shown in Figure 2(b); CYBRD1-positive cells increased with the grade. WHO IV samples yielded the most CYBRD1-positive cells (Figure 2(b)). Consistently, CYBRD1 mRNA expression is shown to be dramatically upregulated within glioma tissue samples and increased with an increasing grade (Figure 2(c)).

### 3.4. *In Vitro* Effects of CYBRD1 on Glioma Cell Phenotype

**Figure 3:**
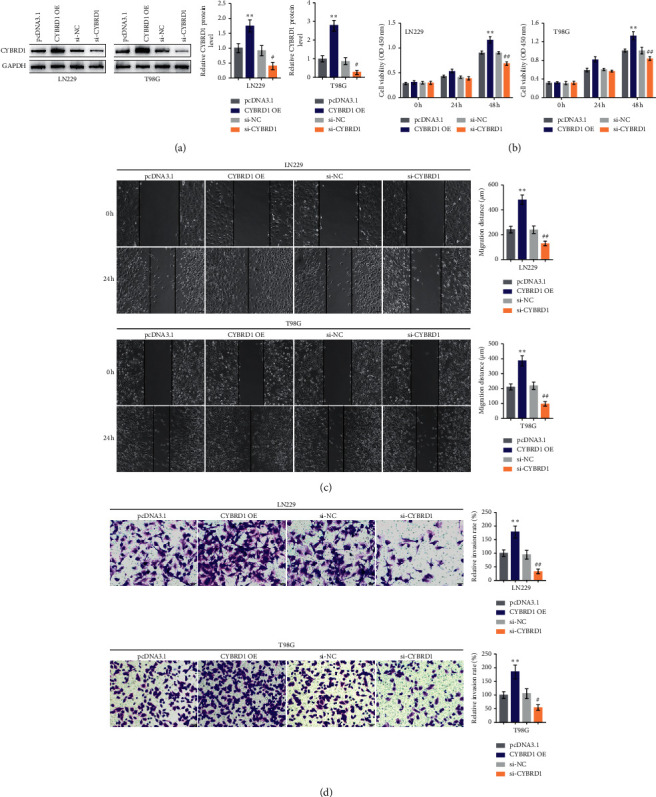
*In vitro* effects of CYBRD1 on glioma cell phenotype. (a) CYBRD1 overexpression or silencing was achieved in glioma LN229 and T98G cells by transfecting pcDNA3.1/CYBRD1 OE or small interfering RNA targeting CYBRD1 (si-CYBRD1); pcDNA3.1 or si-NC was transfected as a negative control. The transfection efficiency was verified by RT-qPCR. Then, LN229 and T98G cells were transfected with CYBRD1 OE or si-CYBRD1 and examined for cell viability by CCK-8 assay (b); cell migration by wound healing assay (c); cell invasion by transwell assay (d). ^∗∗^*p* < 0.01, ^#^*p* < 0.05, ^##^*p* < 0.01.

It was confirmed that CYBRD1 was abnormally upregulated within glioma; CYBRD1 overexpression or silencing was achieved in glioma LN229 and T98G cells to investigate its specific effects on cell phenotype. CYBRD1 overexpression or silencing was achieved by transfecting pcDNA3.1/CYBRD1 OE or small interfering RNA targeting CYBRD1 (si-CYBRD1); pcDNA3.1 or si-NC was transfected as a negative control. The transfection efficiency was verified by RT-qPCR (Figure 3(a)). In both LN229 and T98G cell lines, CYBRD1 overexpression promoted cell viability (Figure 3(b)), migratory ability as revealed by wound healing assay (Figure 3(c)), and invasive ability as revealed by Transwell assay (Figure 3(d)); on the contrary, CYBRD1 silencing attenuated glioma cell aggressiveness (Figures 3(b)–3(d)). The data indicate that glioma cells could harbor a more aggressive phenotype due to CYBRD1 overexpression.

### 3.5. Gene Ontology (GO) Term Enrichment Analysis upon CYBRD1 Co-Expression Genes

**Figure 4:**
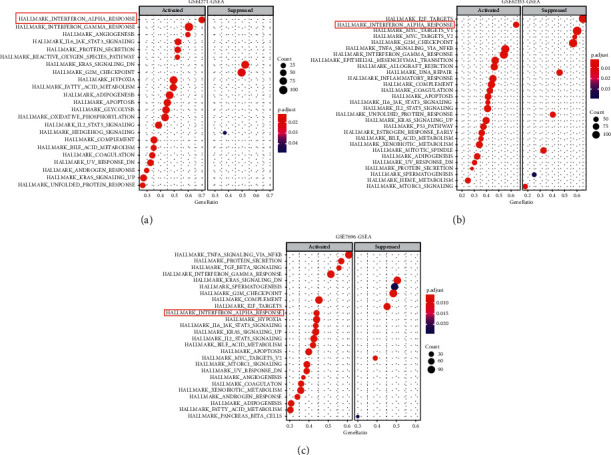
Gene ontology (GO) term enrichment analysis on CYBRD1 co-expressed genes. (a) GO enrichment analysis was performed on CYBRD1 co-expressed genes in GSE4271. (b) GO enrichment analysis was performed on CYBRD1 co-expressed genes in GSE62153. (c) GO enrichment analysis was performed on CYBRD1 co-expressed genes in GSE7696.

To further investigate the functional mechanism of CYBRD1, CYBRD1 co-expressed genes were identified in GSE4271, GSE62153, and GSE7696, respectively. Cases from GSE4271, GSE62153, and GSE7696 were divided by the median value of CYBRD1 expression into high-CYBRD1 and low-CYBRD1 groups, respectively; genes that significantly correlated with CYBRD1 (positively or negatively) were identified and selected for the GO enrichment analysis. Results from all the three data sets indicated that CYBRD1 co-expressed genes were significantly enriched in the adaptive immune response, especially in response to the interferon-alpha (IFN-*α*) pathway (Figure 4). These data suggest that CYBRD1 could potentially participate in glioma cells' response to IFN-*α*.

### 3.6. CYBRD1 Overexpression Reversed the Functions of IFN-*α* upon Glioma Cells

**Figure 5:**
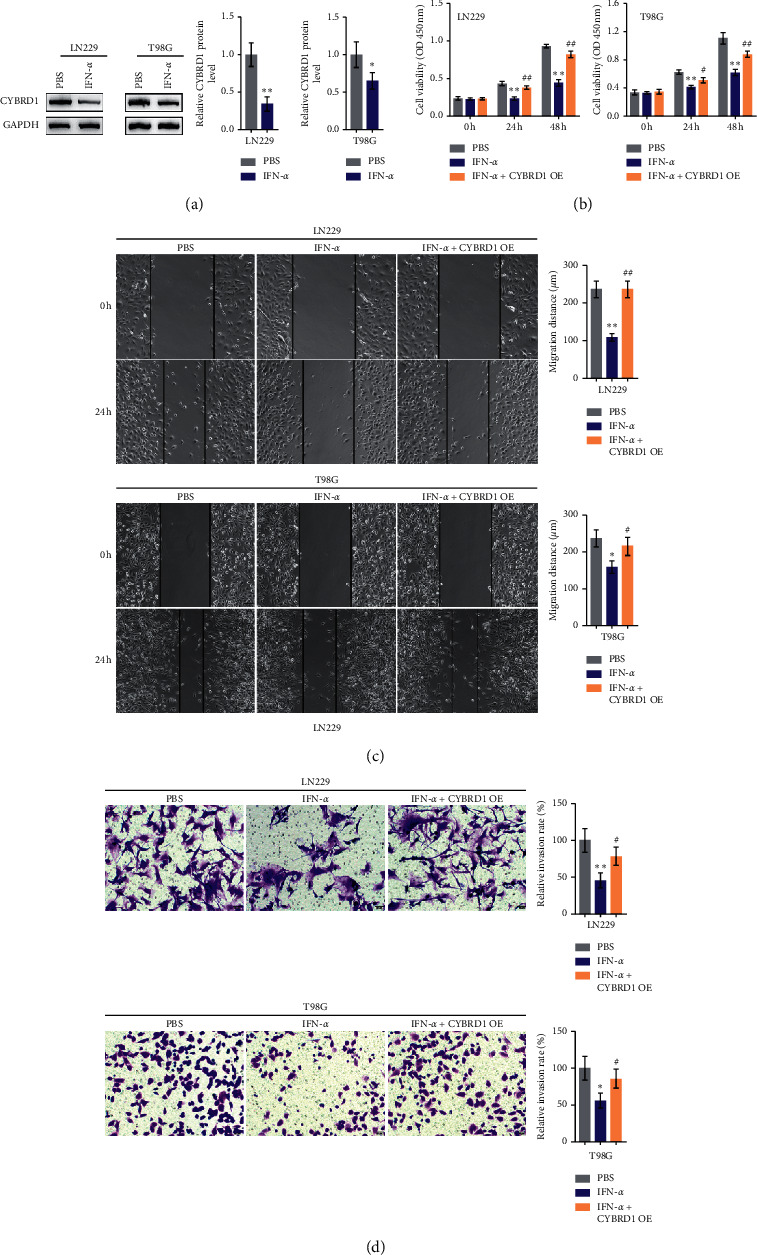
CYBRD1 overexpression reversed the effects of IFN-*α* on glioma cells (a) LN229 and T98G cells were stimulated with IFN-*α* (2000 U/ml) for 24 h and examined for the protein levels of CYBRD1 by immunoblotting. Then, LN229 and T98G cells were transfected with CYBRD1 OE, stimulated with IFN-*α* (2000 U/ml) for 24 h, and examined for cell viability by CCK-8 assay (b); cell migration by wound healing assay (c); cell invasion by transwell assay (d)^*∗*^*p* < 0.05, ^∗∗^*p* < 0.01, compared with the control group (PBS); ^#^*p* < 0.05, ^##^*p* < 0.01, compared with the IFN-*α* group.

To investigate whether CYBRD1 was participated in the response of glioma cells to IFN-*α*, LN229, and T98G cells were stimulated with IFN-*α* (2000 U/ml) for 24 h and CYBRD1 proteins were examined. As depicted in Figure 5(a), IFN-*α* treatment significantly decreased CYBRD1 proteins within these two cells. LN229 and T98G cells were subsequently transfected with CYBRD1 OE. These cells were then stimulated with IFN-*α* (2000 U/ml) for 24 h and examined for glioma cell phenotype. IFN-*α* significantly inhibited glioma cell viability (Figure 5(b)), migratory ability as revealed by the wound healing assay (Figure 5(c)), and invasive ability as revealed by the Transwell assay (Figure 5(d)); after overexpressing CYBRD1, IFN-*α*-repressed cell viability, migration, and invasion were reversed (Figures 5(b)–5(d)). These data indicate that CYBRD1 overexpression could attenuate glioma cell response to IFN-*α*.

### 3.7. CYBRD1 Overexpression Reversed the Functions of IFN-*α In Vivo*

**Figure 6:**
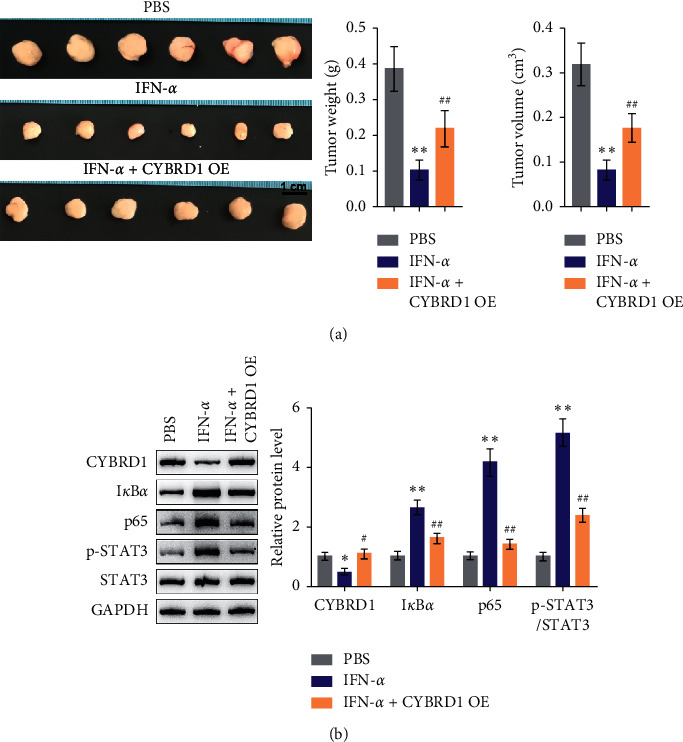
*In vivo* effects of CYBRD1 on xenograft tumor growth in mice model, xenograft tumor models were established in mice by subcutaneous injection of LN229 cells as described. Intratumor injection with IFN-*α* and CYBRD1 overexpression lentivirus was performed. (a) Tumor weight and tumor volume were examined. (b) The protein levels of CYBRD1, I*κ*B*α*, p-STAT3, and STAT3 were examined using immunoblotting.

Finally, xenograft tumor models were established in mice through subcutaneous injection of LN229 cells. Intratumor injection with IFN-*α* or CYBRD1 overexpression lentivirus was performed after xenograft tumor appeared. As shown in Figure 6(a), when compared to the PBS group, the IFN-*α* treatment significantly decreased the weight and volume of xenograft tumor; compared to the IFN-*α* treatment group, CYBRD1 overexpression increased the weight and volume of the xenograft tumor. Regarding the downstream signaling, IFN-*α* treatment significantly decreased the CYBRD1 level and increased the I*κ*B*α*, p65, and p-STAT3 levels compared to the PBS group; compared to the IFN-*α* treatment group, CYBRD1 overexpression increased the CYBRD1 level and decreased the I*κ*B*α*, p65, and p-STAT3 levels (Figure 6(b)). These results indicate that CYBRD1 overexpression could attenuate the inhibitory effects of IFN-*α* on xenograft tumor growth *in vivo*.

## 4. Discussion

Herein, this study confirmed the abnormal upregulation of CYBRD1 in high-grade and recurrent gliomas. Glioma patients with higher CYBRD1 expression predicted poorer survival. CYBRD1 overexpression in glioma cell lines was enhanced, whereas CYBRD1 silencing attenuated the aggressiveness of glioma cells. According to the GO enrichment analysis, CYBRD1 co-expressed genes were enriched in immune responses, particularly in the cell response to IFN-*α*. In IFN-*α*-treated glioma cells, IFN-*α* considerably repressed the viability, migratory ability, and invasive ability of glioma cells, whereas CYBRD1 overexpression attenuated the effects of IFN-*α* treatment on glioma cell aggressiveness.

In 2001 [18], CYBRD1 was identified as belonging to the class of cytochromes b561 that constituted a class of intrinsic transmembrane proteins containing two heme molecules [26, 27]. Additionally, CYBRD1 expression contributed to the maintenance of extracellular ascorbate levels [28]. To meet the high iron demand, the transferrin receptor, a major component of the route for cellular iron intake, is upregulated in many types of cancer cells [29, 30]. Conclusively, CYBRD1 upregulation has been reported in several cancers. It has been revealed by Boult et al. that during Barrett's metaplasia into adenocarcinoma, overexpression of CYBRD1 was observed, which showed to be linked to enhanced iron deposition [20]. In addition, Brookes et al. related higher expression of CYBRD1 and other iron import proteins with an increased risk of colorectal cancer [21]. Moreover, upregulation of CYBRD1 mRNA was observed in breast cancer cell lines MCF, T-47D, BT-474, and ZR-75–30, as well as in prostate cancer cell line DU-145 with approximately 2- to 7-fold induction [31]. In this study, the abnormal upregulation of CYBRD1 in high-grade and recurrent gliomas was reported by bioinformatics and experimental analyses. Glioma patients with higher CYBRD1 expression significantly predicted shorter survival, suggesting that CYBRD1 might be considered a marker possibly involved in the recurrence and prognosis of gliomas.

Although the potential of CYBRD1 as a biomarker has been suggested, its specific effects on cancer cell aggressiveness remain unclear as of yet. Zheng et al. [32] performed bioinformatics analysis, and a ceRNA network was subsequently constructed in glioblastoma with the differentially expressed RNAs and differentially expressed genes. It was found that high levels of *ENSG00000203739* or *ENSG00000271646* expression could potentially suppress miR-637 and promote the expression of possible oncogene CYBRD1, thus promoting the proliferative and invasive capacities of glioblastoma. However, their findings were limited to silicon analyses. Herein, the study first found that CYBRD1 overexpression within glioma cells significantly promoted cell viability, migration, and invasion. On the contrary, CYBRD1 silencing in glioma cells inhibited glioma cell aggressiveness by repressing cell viability, migratory ability, and invasive ability. These *in vitro* findings showcase the oncogenic effect of CYBRD1 overexpression on glioma. In other words, higher CYBRD1 expression might be correlated with more aggressive glioma cell phenotypes.

In order to get a better understanding of the oncogenic role of CYBRD1 in glioma, CYBRD1 co-expression genes were identified by making use of data from TCGA-GBMLGG. By further GO enrichment annotation, these CYBRD1 co-expression genes were found to be significantly enriched in immune response, particularly in the cell response to IFN-*α*. Notably, one of the defining characteristics of glioblastoma is its profound local and systemic immunosuppression. IFN-*α* acts as an important effector molecule involved in various immune responses [33]. To this day, although all trials investigating type I interferon IFN-*α*-based immunotherapies in glioma have not been yielding beneficial results, IFN-*α* has been reported to resensitize TMZ-resistant glioma cells to TMZ [34–37]. For example, Ni et al. [36] reported that the combination of IFN-*α* with TMZ significantly prolonged the survival of mice with orthotopic GSC-1 glioma. Herein, IFN-*α* treatment dramatically suppressed the viability, migratory ability, and invasive ability of glioma cells, whereas CYBRD1 overexpression attenuated IFN-*α*-induced inhibition upon the aggressiveness of glioma cells. These data suggest that CYBRD1 could potentially modulate glioma cell response to IFN-*α*. The *in vitro* results were further ascertained by *in vivo* experiments performed on the xenograft tumor models in mice. IFN-*α* treatment decreased the xenograft tumor weight and volume in mice, whereas CYBRD1 overexpression attenuated the inhibitory effects of IFN-*α* on xenograft tumor growth.

In conclusion, CYBRD1 could potentially serve as a biomarker for glioma recurrence; in other words, higher CYBRD1 expression might be a predictor of earlier recurrence. CYBRD1 overexpression enhances glioma cell aggressiveness and attenuates glioma cell response to IFN-*α*. Previous pathological diagnosis of glioma was dependent on histology. The rapid development of molecular typing and precision medicine in recent years has provided novel directions for early diagnosis and treatment regimens of glioma. Our present findings suggest that higher CYBRD1 expression might be correlated with more aggressive glioma cell phenotypes and a predictor of earlier recurrence. Therefore, CYBRD1 expression level might be a potential biomarker for earlier recurrence and a promising target for glioma therapy. Since the speculation is based on in vitro cell model experiments and in vivo mice model experiments, further in vivo and clinical investigations are much required.

## Data Availability

All data generated or analysed during this study are included in this published article.

## Supplementary Materials

Supplementary Materials Figure S1: selection of upregulated genes in high-grade or recurrent gliomas.
