# Supplementary figures and images for: Highly Expressed CYBRD1 Associated with Glioma Recurrence Regulates the Immune Response of Glioma Cells to Interferon
Source: Evid Based Complement Alternat Med. 2021 Jul 16;2021:2793222. doi: 10.1155/2021/2793222 (PMC8302377; doi:10.1155/2021/2793222)

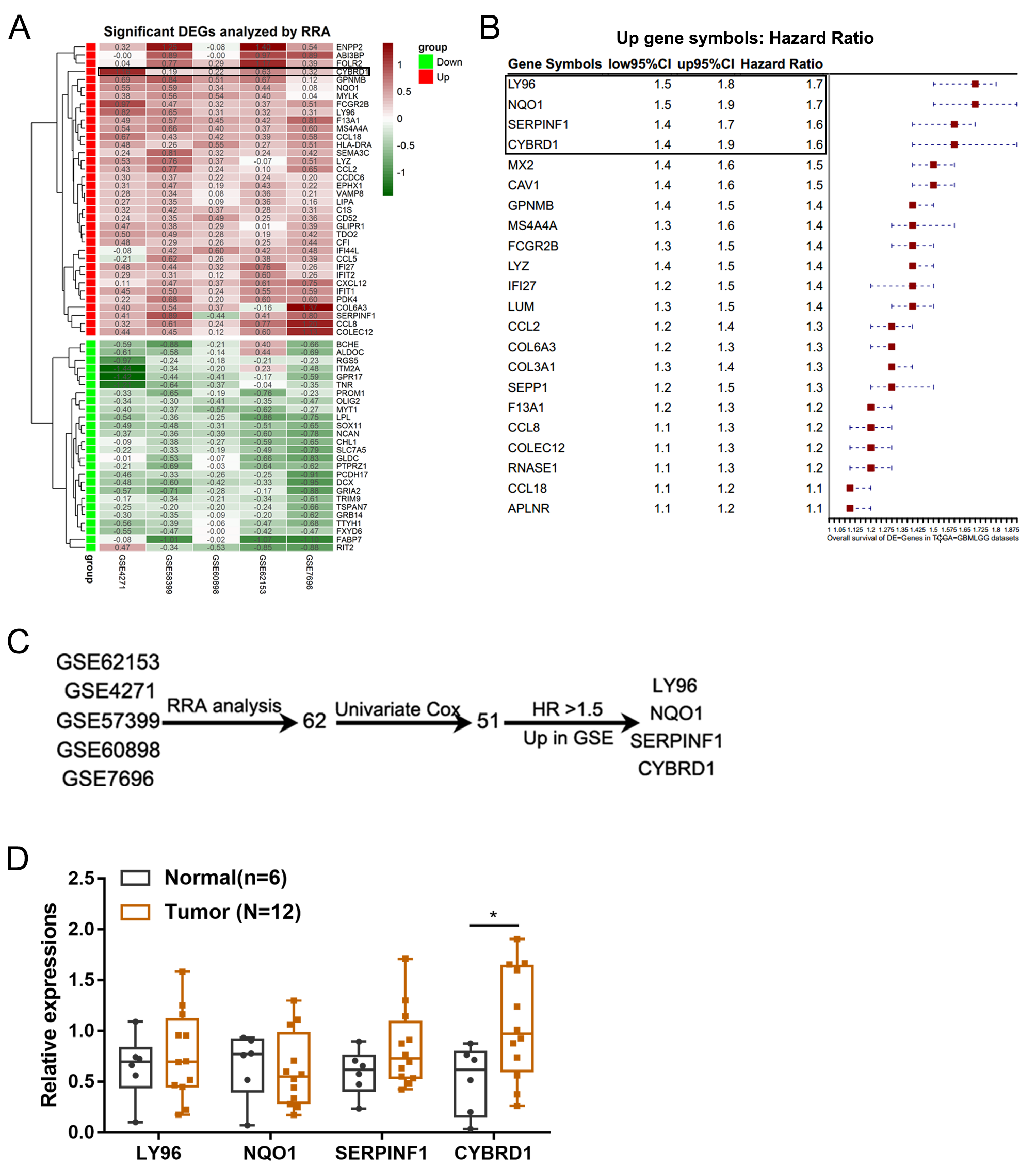

Supplement: Supplementary Materials — Figure S1: selection of upregulated genes in high-grade or recurrent gliomas. [file 2793222.f1.tif]
